# Aberrant expression of melanoma-associated antigen-D2 serves as a prognostic indicator of hepatocellular carcinoma outcome following curative hepatectomy

**DOI:** 10.3892/ol.2014.2823

**Published:** 2014-12-23

**Authors:** RYOJI HASHIMOTO, MITSURO KANDA, HIDEKI TAKAMI, DAI SHIMIZU, HISAHARU OYA, SOKI HIBINO, YUKIYASU OKAMURA, SUGURU YAMADA, TSUTOMU FUJII, GORO NAKAYAMA, HIROYUKI SUGIMOTO, MASAHIKO KOIKE, SHUJI NOMOTO, MICHITAKA FUJIWARA, YASUHIRO KODERA

**Affiliations:** Department of Gastroenterological Surgery (Surgery II), Nagoya University Graduate School of Medicine, Nagoya, Aichi 466-8550, Japan

**Keywords:** hepatocellular carcinoma, expression, prognosis, melanoma-associated antigen-D2

## Abstract

Hepatocellular carcinoma (HCC) is the most common cause of cancer-related mortality globally. Since the prognosis of advanced HCC patients is extremely poor, the development of novel molecular targets for diagnosis and therapy is urgently required. In the present study, the expression of the melanoma-associated antigen-D2 (*MAGE-D2*) gene was investigated to determine whether it affects the malignant phenotype of HCC and thus, may serve as a marker of prognosis. Therefore, the expression of *MAGE-D2* mRNA and MAGE-D2 protein in nine HCC cell lines and 151 pairs of surgical tissues was analyzed. mRNA expression levels were analyzed using reverse transcription-quantitative polymerase chain reaction and immunohistochemistry was used to compare the clinicopathological parameters of the tumors. A significant difference in the level of *MAGE-D2* expression was observed between the normal liver and chronic hepatitis tissues, however, no significant differences were identified among the levels of the chronic hepatitis, cirrhosis and HCC tissues. The expression patterns of the MAGE-D2 protein were consistent with those of its mRNA. The expression levels of *MAGE-D2* mRNA in 66 of 151 (44%) patients were higher in the HCC tissues compared with the corresponding non-cancerous tissues. In addition, the disease-specific survival time was significantly shorter for patients with higher levels of *MAGE-D2* mRNA expression. Multivariate analysis identified increased expression of *MAGE-D2* mRNA as an independent prognostic factor for disease-specific survival (hazard ratio, 2.65; 95% confidence interval, 1.43–4.98; P=0.002). However, increased expression levels of *MAGE-D2* mRNA were not significantly associated with other clinicopathological parameters, including extrahepatic recurrence. These results indicated that *MAGE-D2* mRNA affects tumor progression and may serve as a prognostic indicator following curative resection. In addition, MAGE-D2 may provide a target for the therapy of HCC.

## Introduction

Hepatocellular carcinoma (HCC) is the sixth most common type of cancer and the third leading cause of cancer-related mortality worldwide (1). Recently, the incidence of HCC has rapidly increased and thus, the disease has received considerable attention. Patients diagnosed with HCC often exhibit an adverse outcome due to the aggressive nature of the disease, and surgical resection is usually only effective at the early stages of the disease (2). However, ~70% of these patients develop recurrent tumors within five years (3,4). Even with the advent of the multikinase inhibitor, sorafenib, prolonged survival is limited (5,6). Thus, the development of novel molecular targets for the diagnosis and therapy of HCC are urgently required.

Cirrhosis is the underlying liver disease in 80% of patients with HCC, which distinguishes these tumors from other solid neoplasms (7). Although the high prevalence of hepatitis C virus infection is the main cause of the increasing incidence of HCC, as observed in Western countries (3,8), other etiologies may lead to liver damage and a subsequent increase in HCC incidence, including chronic viral hepatitis B infection, alcohol consumption and exposure to aflatoxin (2). Therefore, clinical approaches for treating HCC are complex and must contend with high molecular variability. Previous studies investigating the molecular mechanisms of carcinogenesis have revealed that the development and progression of HCC is caused by an accumulation of genetic changes, which alter the expression of genes that promote malignant transformation (9–11). The development of novel genomic technologies, such as microarrays and next-generation sequencing, led to the identification of numerous genetic alterations in HCC; however, their clinical significance and the functions of the mutated genes remain largely unclear (2,12).

Current studies have focused on the expression of genes encoding tumor-specific antigens and their association with tumorigenesis and progression (13). Melanoma-associated antigens (MAGEs) represent tumor-specific antigens, which have been increasingly utilized as therapeutic targets for immunotherapy (14). MAGE proteins are classified into types I and II (13,15,16). Type I *MAGE* genes are located on the X-chromosome and include *MAGE*-A, B and C, which are expressed during germ cell development, but not by mature somatic cells. By contrast, the localization, expression and oncological functions of type II MAGE proteins, which include *MAGE*-D, E, F, G and H, are less clear (13,17). Our previous study analyzed the expression of *MAGE-D4* in HCC and esophageal cancer and found that the overexpression of *MAGE-D4* was significantly associated with the malignant phenotypes of these cancers (18,19). However, little is known with regard to the oncological functions of other *MAGE-D* genes. Since melanoma-associated antigen-D2 (MAGE-D2) is involved in cell adhesion (17), we hypothesized that *MAGE-D2* and *MAGE-D4* contribute to the progression of HCC. The aim of the present study was to evaluate the clinical significance of *MAGE-D2* expression in HCC.

## Materials and methods

### Ethics

This study complied with the ethical guidelines of the World Medical Association Declaration of Helsinki Ethical Principles for Medical Research Involving Human Subjects 3(Seoul, Korea; 2008). Written informed consent was obtained from all patients and the study was approved by the Institutional Review Board of Nagoya University (Nagoya, Japan; approval no. 2013-0295-2).

### Sample collection

A total of nine HCC cell lines (Hep3B, HepG2, HLE, HLF, HuH1, HuH2, HuH7, PLC/PRF/5 and SK-Hep1), which were obtained from the American Type Culture Collection (Manassas, VA, USA), were stored at −80°C in Cell Banker^®^ preservative solution (Mitsubishi Chemical Medience Corporation, Tokyo, Japan) and cultured in RPMI-1640 medium (Sigma-Aldrich, St. Louis, MO, USA) supplemented with 10% fetal bovine serum at 37°C in an atmosphere containing 5% CO_2_. Primary HCC tissues and corresponding non-cancerous tissues were collected consecutively from 151 patients undergoing liver resection for the treatment of HCC at Nagoya University Hospital (Nagoya, Japan) between January 1998 and January 2012. Specimens were classified histologically according to the Union for International Cancer Control tumor-node-metastasis classification (seventh edition) (20). Furthermore, background liver status, Child-Pugh classification, hepatitis virus infection status, pre-operative serum tumor markers, tumor multiplicity and maximum size, and pathological observations, including tumor differentiation and vascular invasion, were analyzed. Post-operative follow-up included physical examination, measurement of serum tumor markers every three months, and enhanced chest and abdominal computed tomography examinations every six months. Treatment following recurrence included surgery, radiofrequency ablation, transcatheter arterial chemoembolization and chemotherapy, which was selected according to tumor status and liver function. Tissue samples were immediately flash-frozen in liquid nitrogen and stored at −80°C until RNA was extracted (mean, 28 days). RNA was extracted from tumor samples, which were ~5-mm^2^, without necrotic components and were confirmed to contain >80% tumor cells. Corresponding non-cancerous liver tissue samples from the respective patients were collected >2 cm from the tumor edge, and did not contain any regenerative or dysplastic nodules (12).

### Reverse transcription-quantitative polymerase chain reaction (RT-qPCR)

The expression of *MAGE-D2* mRNA was analyzed using RT-qPCR. Total RNA (10 μg) was isolated from each of the nine aforementioned HCC cell lines, the 151 primary HCC tissues and the corresponding non-cancerous tissues, and used as templates to obtain cDNA. The PCR primer sequences for *MAGE-D2* were as follows: Sense, 5′-TAGAGAAGGCAGACGCATCC-3′ in exon 1 and antisense, 5′-AAGCGAGTTAGACCTGCACC-3′ in exon 2, which amplify a 110-bp sequence. RT-qPCR was performed using nine HCC cell lines and 151 pairs of clinical samples, as well as samples without templates, which served as negative controls, with the SYBR-Green PCR core reagents kit (Perkin-Elmer, Applied Biosystems, Foster City, CA, USA). The SYBR-Green emission intensity was detected using an ABI StepOnePlus Real-Time PCR System (Perkin-Elmer, Applied Biosystems) under the following conditions: One cycle at 95°C for 10 min, followed by 40 cycles at 95°C for 5 sec and 6°C for 30 sec. The expression of glyceraldehyde-3-phosphate dehydrogenase (*GAPDH*) mRNA was quantified in each sample for standardization. mRNA quantification was calculated using the 2^−ΔΔCT^ method. Biological and technical replicates of the cell lines and HCC tissues were performed in triplicate. The expression level of each sample is presented as the value of *MAGE-D2* divided by that of *GAPDH.* In the tumor tissues, *MAGE-D2* mRNA expression was considered to be increased when mRNA levels were higher than those of the corresponding non-cancerous tissues (21).

### Immunohistochemistry (IHC)

IHC was conducted to investigate the localization of *MAGE-D2* in 40 representative sections of well-preserved HCC tissue. Formalin-fixed, paraffin-embedded tissues were dewaxed in xylene twice for 5 min, rehydrated in a graded alcohol series (100, 90 and 70%) followed by H_2_O for 2 min each, then treated with 3% H_2_O_2_ to inhibit endogenous peroxidase activity. Next, epitope retrieval was performed by subjecting samples to five incubations with 10 mM citrate buffer at 95°C for 5 min each. The samples were incubated with Histofine^®^ SAB-PO (Nichirei Biosciences. Inc., Tokyo, Japan) for 5 min to limit non-specific reactivity, then incubated for 1 h at room temperature with a rabbit polyclonal antibody against MAGE-D2 (cat. no. HPA031573; Atlas Antibodies, Stockholm, Sweden), which was diluted (1:500) in antibody diluent (Dako, Glostrup, Denmark). Sections were developed for 2 min using liquid 3,3′-diaminobenzidine substrate (Nichirei Biosciences, Inc.). The staining patterns of the HCC and corresponding non-cancerous tissues were compared. Specimens were randomized and coded prior to analysis by two independent observers who were blinded to the status of the samples. Each observer evaluated all specimens at least twice within a specific time interval to decrease intra-observer variation (21).

### Statistical analysis

The relative mRNA expression levels (*MAGE-D2*/*GAPDH*) between two groups were compared using the Mann-Whitney U test. The χ^2^ test was used to analyze the association between the expression and methylation status of *MAGE-D2* and the clinicopathological parameters. Overall and disease-free survival rates were calculated using the Kaplan-Meier method, and the difference in survival curves was analyzed using the log-rank test. Multivariable regression analysis was performed to detect prognostic factors using the Cox proportional hazards model, and variables with P<0.05 were entered into the final model. All statistical analyses were performed using JMP^®^ 10 software (SAS Institute Inc., Cary, NC, USA). P<0.05 was considered to indicate a statistically significant difference.

## Results

### *MAGE-D2* mRNA expression in HCC cell lines and clinical tissues

**Figure 1:**
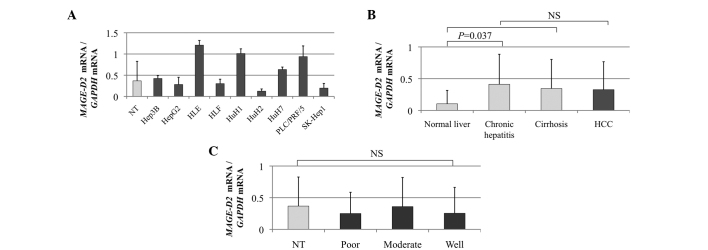
(A) Quantitative polymerase chain reaction analysis of *MAGE-D2* mRNA expression in HCC cell lines and controls (median value of non-cancerous liver tissues). Increased *MAGE-D2* expression was detected in HLE, HuH1, HuH7 and PLC/PRF/5 cells compared with the controls. (B) The *MAGE-D2* mRNA expression level was elevated in liver tissues of patients with chronic hepatitis compared with normal liver tissues, however, no significant differences were identified between patients with chronic hepatitis or cirrhosis. The mean expression level of *MAGE-D2* mRNA was equivalent between HCC and non-cancerous tissues. (C) The mean expression level of *MAGE-D2* mRNA was independent of tumor differentiation. NS, not significant; NT, non-cancerous tissues; MAGE-D2, melanoma-associated antigen-D2; HCC, hepatocellular carcinoma; GAPDH, glyceraldehyde 3-phosphate dehydrogenase.

The heterogeneity of *MAGE-D2* expression in the HCC cell lines was determined using qPCR analysis (Fig. 1A). The *MAGE-D2* mRNA expression levels were compared in the non-cancerous tissues categorized by the background liver status as follows: Normal liver (n=10), chronic hepatitis (n=87), cirrhosis (n=54) and HCC tissues. A significant difference was observed between normal liver and chronic hepatitis tissues (P=0.037), whereas chronic hepatitis and cirrhosis were comparable, indicating that *MAGE-D2* expression was stimulated by chronic inflammation, but not fibrosis (Fig. 1B). The expression level of *MAGE-D2* mRNA in 66 (44%) of the 151 patients was higher in the HCC tissues compared with the corresponding non-cancerous tissues. However, no significant difference in the mean expression level of *MAGE-D2* mRNA was identified between the non-cancerous and HCC tissues (Fig. 1B), indicating that the upregulation of *MAGE-D2* expression is not involved in hepatocarcinogenesis. Furthermore, *MAGE-D2* mRNA expression levels were independent of tumor differentiation (Fig. 1C).

### IHC

**Figure 2:**
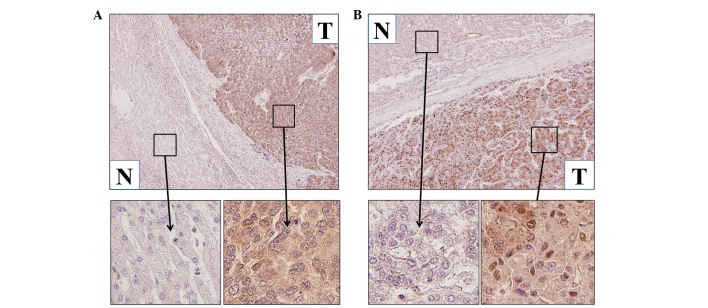
Immunohistochemical analysis of *MAGE-D2* expression in representative patients with HCC. (A) Well-differentiated HCC with cirrhosis and (B) poorly-differentiated HCC with chronic hepatitis. MAGE-D2 was expressed at increased levels in cancerous tissues compared with adjacent non-cancerous tissue cells (original image, ×100 magnification; enlarged areas, ×400 magnification). N, non-cancerous tissue cells; T, tumor tissue cells; MAGE-D2, melanoma-associated antigen-D2; HCC, hepatocellular carcinoma.

The expression of MAGE-D2 protein was determined using IHC in 30 cases exhibiting relative overexpression, underexpression or equivalent *MAGE-D2* mRNA expression in the HCC tissues compared with the corresponding non-cancerous tissues. Two representative cases with high expression levels of *MAGE-D2* mRNA in HCC tissues showed increased expression of MAGE-D2 in the cytoplasm and the nuclei of tumor cells compared with the adjacent non-cancerous tissues (Fig. 2A and B). The results of immunohistochemical staining were consistent with the RT-qPCR data.

### Prognostic value of MAGE-D2 expression in 151 HCC patients

**Figure 3:**
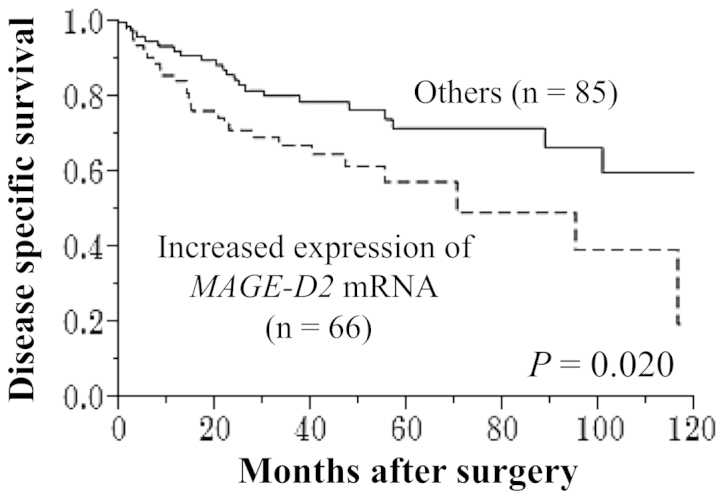
Kaplan-Meier survival curves for 151 patients categorized according to increased expression of *MAGE-D2* mRNA in hepatocellular carcinoma tissues. Disease-specific survival was significantly shorter in patients with increased expression of *MAGE-D2*. MAGE-D2, melanoma-associated antigen-D2.

Increased expression of *MAGE-D2* mRNA was detected in the tumor samples from 66 of the 151 (44%) patients with HCC. The disease-specific survival rate was significantly reduced in the patients with increased expression of *MAGE-D2* mRNA (five-year survival rate, 58 vs. 72%; P=0.020; Fig. 3). The *MAGE-D2* expression level was not associated with recurrence-free survival. Univariate analysis for disease-specific survival showed that advanced age, α-fetoprotein levels of >20 ng/ml, protein induced by vitamin K antagonists II levels of >40 mAU/ml, multiple tumors, a tumor size of ≥3.0 cm, serosal infiltration, vascular invasion, positive margin status and increased expression of *MAGE-D2* mRNA were all significant prognostic indicators of adverse outcomes. Multivariate analysis identified increased expression of *MAGE-D2* mRNA as an independent prognostic factor for disease-specific survival (hazard ratio, 2.65; 95% confidence interval, 1.43–4.98; P=0.002; Table I). Increased expression of *MAGE-D2* mRNA was not significantly associated with other clinicopathological parameters, including extrahepatic recurrence.

## Discussion

Extensive studies and the development of novel genomic technologies may improve our understanding of the molecular pathogenesis of HCC (21–23). Gene signatures derived from tumors and corresponding non-cancerous tissues may identify patients who are at high risk of developing HCC and would benefit from potential chemopreventive strategies (24,25).

MAGE-D2 is encoded by one of the cancer testis family of genes and is located on chromosome Xp11.21 (26,27). In contrast to the testis- and tumor-specific expression of numerous MAGE type I genes, *MAGE-D2* mRNA is expressed in healthy human tissues and the majority of cell types that have been examined (28,29). In previous studies, *MAGE-D2* expression has been analyzed in a clinical setting using high-density oligonucleotide DNA arrays and served as a marker to predict the occurrence of liver metastases from colorectal tumors (30,31). The function of MAGE-D2 is unclear, however, its increased expression may promote the cancer cell adhesion to the vascular epithelium (17). Since MAGE-D2 protects melanoma cells from tumor necrosis factor-related apoptosis-inducing ligand (TRAIL)-induced apoptosis, this observation is of particular note, as TRAIL is involved in the killing of melanoma cells by the immune system and is expressed by a number of immune cells, including activated CD4 and CD8 T lymphocytes, natural killer cells and dendritic cells (32). To the best of our knowledge, the present study is the first to evaluate *MAGE-D2* expression in HCC.

In the present study, once the direct correlation between the expression patterns of *MAGE-D2* mRNA and MAGE-D2 had been identified, the clinical significance of *MAGE-D2* expression was evaluated using RT-qPCR. Increased expression of *MAGE-D2* mRNA in HCC tissues was found to significantly correlate with an adverse outcome and was identified as one of the independent prognostic factors after curative hepatectomy. However, future studies using a larger patient cohort are required to confirm these observations. These results question whether the aberrant expression of *MAGE-D2* contributes to the carcinogenesis and progression of HCC. Although the present study indicated that *MAGE-D2* expression is modulated by chronic inflammation, the expression levels of *MAGE-D2* in HCC and the corresponding non-cancerous tissues were equivalent, indicating that the upregulation of *MAGE-D2* was incidental in hepatic carcinogenesis. By contrast, increased expression levels of *MAGE-D2* were significantly associated with earlier mortality following curative resection, indicating that the upregulation of *MAGE-D2* contributed to the progression of HCC rather than to carcinogenesis.

The use of cDNA microarrays has identified *MAGE-D2* expression as a predictor of the metastatic potential of colorectal cancer (31). By contrast, in the present study, no significant correlation was identified between the expression pattern of *MAGE-D2* mRNA and extrahepatic recurrence. Therefore, the biological functions of *MAGE-D2* in HCC and colorectal cancer may differ. Notably, the increased expression of *MAGE-D2* demonstrated high prognostic value despite the absence of a significant association with other important prognostic factors, including tumor size, multiplicity, vascular invasion, and advanced stage. This reveals the unique prognostic value of *MAGE-D2* for HCC and indicates that HCC patients with increased expression of *MAGE-D2* must be categorized into a high-risk group with an adverse prognosis even during the early stage of HCC.

The present study was limited by the lack of a functional analysis of MAGE-D2. Future studies, which include pathway analysis in hepatocarcinogenesis and functional analysis, are required to analyze the molecular mechanisms that underlie the biological function of MAGE-D2 in HCC.

In conclusion, the results of this study indicated that *MAGE-D2* mRNA overexpression contributes to tumor progression and thus, may serve as a prognostic indicator following curative resection, as well as a potential therapeutic target in HCC.

## Figures and Tables

**Table I:** Prognostic factors of disease-specific survival in 151 hepatocellular carcinoma patients.

		Univariate			Multivariate		
			
Variables	n	Hazard ratio	95% CI	P-value	Hazard ratio	95% CI	P-value
Age (≥65 years)	84	1.92	1.07–3.57	0.030^a^	1.60	0.87–3.05	0.133
Gender (male)	126	1.27	0.60–3.13	0.553			
Background liver (cirrhosis)	54	1.58	0.88–2.81	0.123			
Pugh-Child’s classification (B)	11	0.93	0.28–2.32	0.889			
α-FP (>20 ng/ml)	70	1.90	1.07–3.42	0.029^a^	1.32	0.69–2.50	0.395
PIVKA II (>40 mAU/ml)	93	2.10	1.14–4.07	0.016^a^	1.20	0.60–2.53	0.610
Tumor multiplicity (multiple)	34	2.09	1.11–3.76	0.023^a^	1.31	0.67–2.48	0.418
Tumor size (≥3.0 cm)	104	2.20	1.13–4.71	0.020^a^	1.37	0.61–3.36	0.453
Tumor differentiation (well)	35	0.55	0.25–1.10	0.095			
Growth type (invasive growth)	24	1.44	0.69–2.76	0.318			
Serosal infiltration	37	2.51	1.32–4.61	0.006^a^	1.47	0.70–3.02	0.304
Formation of capsule	104	1.05	0.57–2.02	0.884			
Infiltration to capsule	83	1.20	0.67–2.18	0.537			
Septum formation	98	0.87	0.49–1.60	0.651			
Vascular invasion	37	3.40	1.87–6.07	<0.001^a^	2.42	1.17–4.97	0.017^a^
Margin status (positive)	28	2.64	1.42–4.73	0.003^a^	2.84	1.48–5.36	0.002^a^
Increased expression of *MAGE-D2* mRNA	66	1.96	1.10–3.54	0.022^a^	2.65	1.43–4.98	0.002^a^

a Statistically significant (P<0.05).

Univariate and multivariate analyses were performed using the log-rank test and the Cox proportional hazards model, respectively. CI, confidence interval; α-FP, α-fetoprotein; PIVKA, protein induced by vitamin K antagonists; MAGE-D2, melanoma-associated antigen-D2.
